# S100 Proteins as Diagnostic and Prognostic Markers in Colorectal and Hepatocellular Carcinoma

**DOI:** 10.5812/hepatmon.7240

**Published:** 2012-10-12

**Authors:** Claudia Maletzki, Peggy Bodammer, Anne Breitrück, Claus Kerkhoff

**Affiliations:** 1 Department of General Surgery, Division of Molecular Oncology and Immunotherapy, University of Rostock, Rostock, Germany; 2 Department of General Surgery, Division of Gastroenterology, University of Rostock, Rostock, Germany; 3 Fraunhofer Institute for Cell Therapy and Immunology, Department of Immunology, AG “Extracorporeal Immune Modulation (EXIM)”, Rostock, Germany; 4 Department of Internal Medicine, Division of Nephrology, University of Rostock, Rostock, Germany

**Keywords:** Biological Markers, Cell Transformation, Neoplastic, Matrix Metalloproteinases

## Abstract

**Context:**

Clinical and experimental studies have suggested a link between S100 gene ex­pression and neoplastic disorders, however, the molecular mechanisms of this associa­tion are not well understood. The aim of this review was to conduct a comprehensive literature search in order to understand the possible underlying molecular mechanisms of this association. We also discuss their application as diagnostic and prognostic mark­ers in colorectal and hepatocellular carcinoma.

**Evidence Acquisitions:**

We searched Pubmed (NLM) and Web of Science (ISI Web of Knowledge).

**Results:**

S100 genes display a complex expression pattern in colorectal and hepatocel­ lular carcinoma. They are expressed in tumor and/or tumor stroma cells, and they exert both pro- and antitumorigenic actions. In view of this complexity, it becomes clear that S100 proteins might act as both friend and foe. The biological role of the S100 genes is predicted to depend on the relative contributions of the different cell types at specific stages of tumor progression.

**Conclusions:**

Further research is required in order to uncover the functional role of S100 genes in tumorigenesis. Answers to this issue are needed before we can more fully un­derstand the clinical relevance of S100 protein expression within epithelial tumors, with regard to their potential applicability as biomarkers for diagnosis and therapy decisions.

## 1. Context

Over the last decade a number of S100 genes have been found to be differentially expressed in cancer cells, by comparative and functional genomics. However, the molecular mechanisms by which they promote tumorigenesis and progression to malignancy remain unclear. In general, S100 overexpression is coupled with; poor tumor differentiation, aggressiveness, advanced stage, and metastatic growth. S100 expression has therefore been considered to be a negative prognosticator for patients´ survival. On the other hand, recent studies have also demonstrated that S100 genes can act as tumor suppressors in some cancer entities. The aim of this review was to conduct a comprehensive literature search to better understand the putative cellular functions of S100 proteins in the context of tumorigenesis. We discuss their complex expression patterns in tumor and stromal cells as well as their pro- and antitumorigenic actions. Furthermore, we present evidence for their application as diagnostic and prognostic markers in colorectal and hepatocellular carcinoma.

## 2. Evidence Acquisition

Pubmed (NLM) and Web of Science (ISI Web of Knowledge) were searched with key words ‘S100 genes’, ‘colorectal carcinoma ‘, ‘hepatocellular carcinoma’, and ‘inflammation associated tumorigenesis’, in the past 10 years.

## 3. Results

We were able to find 161 research and review articles relevant to this topic, either directly or indirectly. From the information given in these papers, we drew out the following aspects.

### 3.1. The S100 Protein Family

The S100 protein family is a multigenic group of nonubiquitous cytoplasmic EF-hand Ca2+-binding proteins, sharing significant structural similarities at both genomic and protein levels. They are differentially expressed in a wide variety of cell types (1) and have been reported to be involved in the regulation of inflammatory responses (2), as well as in the metastasis development of several cancers (3). The S100 protein family comprises 24 known human members each coded by a separate gene. At least 19 of these gene are located on chromosome 1q21. This S100 gene cluster is close to the epidermal differentiation complex (4) as well as to a psoriasis susceptibility region, the PSORS4 locus (5, 6). An additional important indication for their involvement in neoplastic disorders is that the S100 gene cluster is found near a break-point region on human chromosome 1q21 which, if affected, is responsible for a number of genetic abnormalities related to cancer (7-11). S100 proteins are p53 (12-14), NF-κB (15-19) or AP-1/Fos (20-25) target genes, thus playing an important role in inflammation-associated carcinogenesis. Although the function of S100 proteins in cancer cells is still unknown, the specific expression patterns of these proteins are a valuable prognostic tool. Several general articles on S100 proteins have been published recently (26, 27), but in this chapter we will only include details of those functions which have an impact on cancer.

S100 proteins are complex in their actions as they have both intracellular and extracellular functions. In resting cells, S100 proteins are localized intracellular. However, upon cellular activation or exposure to proinflammatory cytokines, they are either specifically released (mainly from cells of the myeloid lineage) or constitutively secreted from epithelial-like cells and tumor cells. Upon their release into the extracellular environment, they exert regulatory effects on several different cell types. S100 proteins can signal this by binding to the receptor for advanced glycation end products (RAGE), toll-like receptors (TLRs), or other receptors.

In addition, S100 proteins interact with multiple molecular targets both in a calcium-dependent and independent manner. Thereby, they regulate multiple cellular pathways that play key roles in tumor progression and metastasis.

#### 3.1.1. Binding With Cytoskeletal Proteins Thereby Increasing Cell Migration

One molecular mechanism by which S100 proteins exert their protumorigenic effect depends on their interaction with cytoskeletal proteins resulting in an enhancement of cell migration. The number of cytoskeletal proteins includes tropomyosin (28, 29), nonmuscle myosin (30-38), actin (39, 40), and tubulin (41-43).

#### 3.1.2. Binding With DNA Binding Factors Thereby Increasing Cell Migration

S100 proteins also modulate cell migration due to their transcriptional activity, either by direct DNA binding, or by interacting with other DNA-binding proteins. For example, S100A4 negatively regulates the expression of Ecadherin (44, 45) or reduces the expression of occludin, which is a tight junction protein (46), thus loosening epithelial cell integrity. Similarly, the overexpression of S100P leads to changes in the expression levels of several cytoskeletal proteins, including cytokeratins 8, 18, and 19. A cellular consequence of this change is a disorganization of the actin cytoskeleton network and changes in the phosphorylation status of the actin regulatory protein, cofilin (47).

#### 3.1.3. Interaction With p53

The tumor suppressor p53 protein is a homotetrameric transcription factor that regulates several cellular processes. In response to stress (ie, DNA strand break), p53 prevents tumorigenic transformation through the induction of cell cycle arrest or apoptosis. The crucial role that this protein plays is reflected in the fact that more than 50% of human cancers contain mutations in this gene (48). In unstressed cells, the level of p53 tumor suppressor is low, however, upon stress challenge; p53 is activated through posttranslational modifications that increase its stability. The regulation of protein stability is one of the most effective mechanisms for controlling the function of p53. Key to this process is MDM2, an E3 ligase that targets p53 for ubiquitination. Several S100 proteins such as S100B (49, 50), S100A1, S100A2 (50, 51), S100A4 (46, 51) , S100A6, and S100A14 (52, 53) have been shown to interact with MDM2 (49). Direct protein-protein interaction thereby promoting its degradation, has been demonstrated in S100B (54) and S100A4 (55). Both processes result in the loss of p53-dependent tumor suppression activities. The consequences are intriguing, connecting the metastasispromoting activities of S100 proteins to the large set of important p53-mediated functions, with broad potential importance in cancer development and metastasis. In addition, S100 proteins also appear to interact with two other members of the p53 family, p63 and p73 (56). It has been discussed that the target preference of each individual S100 protein, their specific expression pattern and their individual calcium dependency, as well as the splicing variation of the p53 family, has to be considered when studying the biological effects of S100 proteins in their biological context. It is noteworthy, that potential p53 binding sites have been identified in the promoter of several S100 genes (see above), indicating that the metastasis- promoting properties of S100 proteins are not as clear-cut as has previously been suggested, this is due to their interaction with p53-dependent apoptosis. This fact may explain why some members of the S100 family are markedly down-regulated in malignant cells, in comparison to normal cells. In addition, it has been reported that S100A4 enhances p53-dependent apoptosis while S100A2 promotes p53 transcriptional activity (57).

#### 3.1.4. S100 Proteins: Role in Degradation of Extracellular Matrix and Metastasis

Degradation of the extracellular matrix is another important factor affecting the motility of cancer cells. Herein, S100 proteins have been ascribed to contribute by various molecular mechanisms. For example, intracellular S100A14 promotes cell motility and invasiveness by regulating the expression and function of matrix metalloproteinase (MMP)-2 in a p53-dependent manner (52, 58). P53 is a transrepressor of MMP2 gene expression. Intracellular S100A14 affects p53 transactivity and stability, resulting in an enhancement of MMP2 gene expression. Extracellular S100A4 binds to RAGE and thereby induces the upregulation of MMP-13 (59, 60), MMP-2 (58), and MMP-9 gene expression (61), allowing cell invasion and thus promoting metastasis formation. S100A8/A9 overexpression also caused the upregulation of MMP-9 in HaCaT keratinocytes (62). Here, MMP9 gene induction depends on NF-κB activation, and intracellular S100A8/A9 was shown to promote epithelial NADPH oxidases and subsequently NF-κB activation. S100P induces the expression of cathepsin D, an aspartyl protease, that takes part in the proteolytic degradation of the extracellular matrix, hence it increases the invasive potential of the tumor (47).

#### 3.1.5. Intra- and Extracellular Localization With Opposite Effects on Cell Growth

Contradictory findings have been reported for the effect of S100 proteins on cell growth. Beside p53 (see above), another checkpoint for cells, either to set about repairing themselves or to commit suicide through apoptosis, is p21/WAF1 also known as cyclin-dependent kinase (CDK) inhibitor 1 or CDK-interacting protein 1. The p21/WAF1 protein functions as a regulator of cell cycle progression at G1 via inhibition of cyclin-CDK2 or -CDK1 activity. The expression of this gene is tightly controlled by p53, through which this protein mediates the p53-dependent cell cycle G1 phase arrest, in response to a variety of stress stimuli. Accumulation of S100A11 in the nucleus has been observed in normal human keratinocytes by high calcium concentrations or TGF-ß exposure, where it induced p21/ WAF1 (63, 64). In contrast, S100A11 has been shown to be overexpressed in many human cancers. This apparent discrepancy has been resolved by the finding that the production and secretion of S100A11 are markedly enhanced in human squamous cancer cells. Extracellular S100A11 binds to RAGE thereby enhancing the production of epidermal growth factor family proteins and this results in growth stimulation (65).

**Figure 1:**
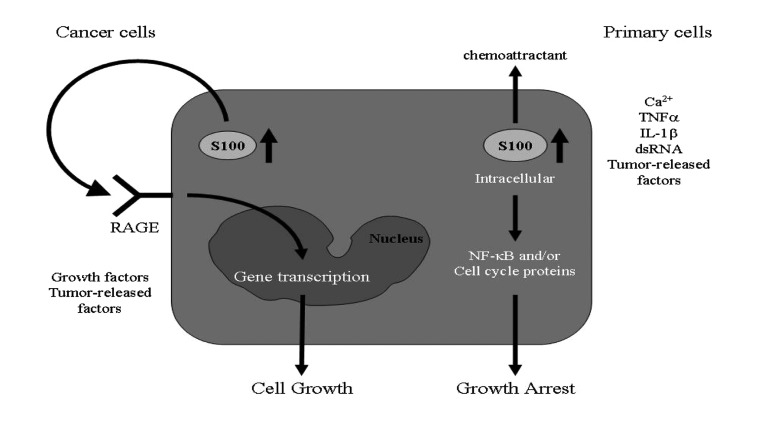
In Resting Epithelial Cells, S100 Proteins are Primarily Localized in the Cytoplasm

In human hepatocellular carcinoma cells (HCC), TGF-ß has been demonstrated to induce growth suppression (66). S100A11 has also been shown to be involved since TGF-ß induces S100A11 gene expression and translocation into the nucleus, where it interacts with p21/Waf1. In addition, S100A8/A9 appears to have both growth-stimulatory and anti-proliferative properties. Extracellular S100A8/ A9 stimulates the proliferation of normal human keratinocytes (67), while intracellular S100A8/A9 functions as a mediator for growth suppression (62). Thus, S100A8/A9 plays an ambivalent role with respect to the growth regulation of keratinocytes, and the biological effects of S100 proteins mediated by intracellular versus extracellular actions, have to be carefully elucidated (Figure 1).

#### 3.1.6. Extracellular S100 Proteins: Interaction With RAGE

Another important feature is that S100 proteins form heteromers, as well as higher order complexes, and these complexes display different affinities to target proteins depending on their oligomerization state. This has been demonstrated for p53 and RAGE (56, 68). Moreover, extracellular S100 proteins bind to various receptors and they also bind to different epitopes on individual receptors. RAGE has been shown to transduce the extracellular effects of S100B, S100A4, S100A6, S100A8/A9, S100A11, S100A12, S100A13, and S100P. For S100B and S100A6, it has been shown that they not only interact with distinct RAGE immunoglobulin domains, but they also exert opposite effects on cell survival. At similar concentrations, S100B increased cellular proliferation, whereas S100A6 triggered apoptosis. In addition, both S100 proteins induced the formation of reactive oxygen species; however, S100B recruited phosphatidylinositol 3-kinase/AKT and NF-κB, whereas S100A6 activated JNK. These data indicate the complexity of S100/RAGE cellular signaling (69).

Another layer of complexity is attributed to the fact that some S100 proteins exert both protumorigenic and antitumorigenic effects. The prosurvival activity of S100B depends on its binding to RAGE, RAGE-induced activation of MEK–ERK1/2–NF-κB pathway, and the up-regulation of anti-apoptotic factor bcl-2 (70). On the other hand, high concentrations of S100B induce apoptotic cell death (70). Remarkably, the bimodal function of S100B is mediated by RAGE engagement. In contrast, S100A8/A9 also exerts both growth-stimulating and apoptosis-inducing activities; however, these cellular effects are mediated by distinct receptors. S100A8/A9 exerts its apoptosis-inducing activity through the mitochondrial pathway since overexpression of the mitochondrial transmembrane deficient form of the proapoptotic BNIP3 (ΔTM-BNIP3) partially reversed the cytotoxicity of S100A8/A9 (71, 72). Further features are a drop in the mitochondrial membrane potential; Bak and BNIP3 activation, selective translocation of Smac/Diablo and Omi/HtrA2, and decreased Drp1 expression. RAGE gene knockdown and RAGE blocking experiments have demonstrated that the apoptosisinducing activity of S100A8/A9 was not mediated by RAGE ligation. However, the growth-promoting activity obvious at low micro molar concentrations was mediated by RAGE (71). In another study, the first evidence which was provided is that S100A8/A9 induces both apoptosis and autophagy. S100A8/A9-induced cell death involves BNIP3 and an increase in reactive oxygen species (ROS) production by the mitochondria, subsequently this is followed by mitochondrial damage and lysosomal activation. These data indicate that ROS is the critical factor that integrates S100-induced mitochondrial and lysosomal death pathways (73).

### 3.2. S100 Gene Expression in Tumor Stroma Cells

Comparative and functional genomics revealed a number of S100 proteins to be differentially expressed in cancer cells. Some of these studies are hampered by the fact that there has not been sufficient discrimination between the expression within the tumor or the tumor stroma cells. Thus, although differential expression has been noted in tumors, the cellular role of S100 proteins in tumorigenesis needs to be defined in the context of their cell-type specific expression pattern. Tumors are heterogeneous organs containing myofibroblasts, endothelial cells, inflammatory cells, vascular cells and other cells (see below), in addition to malignant cells. These tumor-companion cells are intermingled in the tumor-associated stroma that comprises most of the tumor mass in many carcinomas. The stromal microenvironment of tumors is involved in the suppression of immune control, in the promotion of neoangiogenesis, in the proliferation and invasion of carcinoma cells. A dynamically molecular crosstalk takes place between the cancer and stroma cells, mostly in the form of growth factor signaling, and this contributes to niche formation for the metastasis. The relevance of the reactive stroma that surround the cancer cells, is highlighted by the fact that the molecular properties of tumor stroma are predictive of disease outcomes (74). Research, aiming to establish the roles of the tumor-associated stroma in facilitating the spread of carcinoma cells into distant organs, has provided an abundance of data and greater knowledge of the biology of metastatic carcinoma and associated stromal cells (75). This has stimulated further advances in the development of novel therapeutic approaches targeting tumor metastasis. Thus, understanding the roles of S100 proteins in cancer presumes the cell-type(s) in which the S100 proteins are expressed and released, and the molecular mechanisms by which their gene expression is regulated. Finally, the investigation of the cellular responses and the underlying mechanisms, whereby these proteins mediate their biological effects, may offer new therapeutic strategies to prevent, treat, and predict cancer.

#### 3.2.1. Fibrocytes

CD34+ fibrocytes are constitutive elements of the connective tissue, where they play a role in matrix synthesis and tumor-associated stromal remodeling. In colorectal adenocarcinoma (CRC), normal colonic stroma harbor CD34+ fibrocytes, whereas this cell population is absent from the stroma of invasive adenocarcinoma (76). They are a distinct population of leukocytes with the characteristics of fibroblasts. They are capable of producing matrix proteins, such as fibroblast collagen type 1 and 3 (77, 78), and they are fully functional in their presentation of antigens to naive T-cells. Fibrocytes play a prominent role in fibrosis, and they can be discriminated from fibroblasts in fibrotic lesions by a set of specific markers, including S100A8/A9 (79). To date, there has been no data available concerning the cellular role of S100A8/A9, or other S100 proteins in fibrocytes in the context of tumorigenesis.

#### 3.2.2. Dendritic Cells

The clinical significance of tumor-infiltrating dendritic cells (DCs) has been reported in a variety of human solid tumors, as shown by the correlations found between the presence of tumor-infiltrating dendritic cells and the clinical prognosis (80-83). S100A4 (84), S100A6 (84), S100A8 (85), S100A9 (85), S100A12 (86, 87) and S100B (87) have been found in DCs. A significant role in DCs has been shown for S100A8/A9 (85). S100A8/A9 expression is found in both myeloid DCs and plasmacytoid DCs. DCs of S100a9-/- mice promote higher T cell proliferation compared to wild-type DC in mixed lymphocyte reactions, and they express more DC cell surface markers, (CD205, IA) and co-stimulatory molecules (CD40, CD86) compared to wild-type cells after lipopolysaccharide (LPS) stimulation (88). In addition, S100A9 regulates B7 molecule expression and reduces antigen presentation by DCs and subsequent T-cell priming. The absence of S100A9 markedly increases T-cell activation and exacerbates allograft rejection (89).

Consistent with these results, DC differentiation is blocked by S100A9 overexpression (86). The same study showed that up-regulation of S100A8 and S100A9 by tumor- derived factors might represent one major mechanisms by which abnormal DC differentiation is established in cancer (90-93).

#### 3.2.3. Myeloid-Derived Suppressor Cells

Myeloid-derived suppressor cells (MDSCs) are a heterogeneous population of immune cells that accumulate in tumor-bearing hosts, as well as in response to inflammation. In mice, these cells are defined by the surface expression of CD11b and Gr-1 for granulocytic MDSCs or CD11b, Gr 1, CD115, and F4/80 for monocytic MDSCs, respectively (86). To date, only two members of the S100 protein family, S100A8 and S100A9, have been detected in MDSCs. Interestingly, mice lacking S100A9 showed significantly reduced tumor incidence, growth and metastasis, reduced chemokine levels, and reduced infiltration of CD11b+ Gr1+ cells within tumors and pre-metastatic organs. Studies using bone marrow chimeric mice revealed that S100A8/ A9 expression on myeloid cells is essential for the development of colon tumors (94). MDSCs synthesize and secrete S100A8/A9, and the binding of S100A8/A9 not only induces MDSCs via RAGE activation, but it also promotes MDSC migration (95). Furthermore, S100A8/A9+ MDSCs impair tumor immunity by inhibiting T and NK cell activation, by polarizing immunity toward tumor-promoting type 2 phenotypes, and by inhibiting DC differentiation. These responses are considered to be responsible for the limited effectiveness or failure of cancer vaccines and other immunotherapies (85). Tumor cells and Mac-1+myeloid cells utilize a common pathway for their migration to the lung (96). S100A8/A9 also exerts chemotactic activity for Mac-1+ myeloid cells. After preparation of the target tissue to accept the malignant cells, tumor cells mimic Mac-1+ myeloid cells in response to S100A8/A9 chemotactic signaling and they migrate to the lung. These findings suggest that S100A8/A9 is an attractive target for the development of future strategies to counteract the tumor metastasizing to certain organs. In accordance with this suggestion, the suppression of Mac-1+ myeloid cells was shown to enhance tumor response to radiation (97). As mentioned above, S100A4 plays an important role in tumor metastasis, due to its effects on tumor cell migration. Recently, it has been shown that S100A4 is expressed in macrophage chemotaxis (98). S100A4 deficiency affected chemotactic motility of bone marrow macrophages in vitro and impaired recruitment of macrophages to sites of inflammation in vivo. In addition, S100A4-/- bone marrow macrophages formed unstable protrusions, over assembled myosin-IIA, and exhibited altered colony-stimulating factor-1 receptor signaling. This study established S100A4 as a regulator of physiological macrophage motility. It is becoming increasingly clear that S100 proteins have distinct functions in different cell types which are involved in metastasis development of several cancers. Further studies are required to elucidate whether the effects of S100 proteins are mediated by intracellular or extracellular mechanisms and whether they modulate cellular responses in tumor stroma cells and/or tumor cells.

### 3.3. S100 Gene Expression in Tumor Cells

The family of S100 Ca2+-binding proteins is closely related to several tumors, including those of the colon and the liver. Table 1 summarizes the expression of S100 genes in different stages of tumor progression within these two tumor entities.

**Table 1:** S100 Expression in Different Stages of Tumor Progression

	S100 Expression														
**Colorectal Carcinoma (119)**	**A1 (153)**	**A2 (153)**	**A3 (153)**	**A4 (99, 154)**	**A5 (153)**	**A6 (107, 109, 153)**	**A7**	**A8 (109)**	**A9 (109)**	**A10**	**A11 (109, 155)**	**A14 (156)**	**B (112)**	**P (133-135, 140)**	**G**
Normal epithelium	expression at very low level	expression at very low level	expression at very low level	expression at very low level	protein expression verified by IHC and/or WB	protein expression verified by IHC and/ or WB	N.D.	protein expression verified by IHC and/or WB	protein expression verified by IHC and/or WB	N.D.	protein expression verified by IHC and/or WB	protein expression verified by IHC and/ or WB	protein expression verified by IHC and/or WB	expression at very low level	N.D.
Preneoplastic adenomas	expression at very low level	N.D.	N.D.	protein expression verified by IHC and/or WB	reduced/ lost expression compared to normal tissue	protein expression verified by IHC and/ or WB	N.D.	protein expression verified by IHC and/or WB	stage-dependent overexpression	N.D.	protein expression verified by IHC and/or WB	protein expression verified by IHC and/ or WB	protein expression verified by IHC and/or WB	protein expression verified by IHC and/or WB	N.D.
Neoplastic primaries	reduced/ lost expression compared to normal tissue	N.D.	N.D.	overexpression compared to normal tissue	reduced/ lost expression compared to normal tissue	stage-dependent overexpression	N.D.	stagedependent overexpression	stage-dependent overexpression	protein expression verified by IHC and/or WB	stage-dependent overexpression	reduced/ lost expression compared to normal tissue	stagedependent overexpression	stagedependent overexpression	N.D.
Metastatic lesions	reduced/ lost expression compared to normal tissue	N.D.	N.D.	stagedependent overexpression	reduced/ lost expression compared to normal tissue	stage-dependent overexpression	N.D.	stagedependent overexpression	stage-dependent overexpression	N.D.	protein expression verified by IHC and/or WB	expression at very low level	stagedependent overexpression	stagedependent overexpression	N.D.
**Hepatocellular carcinoma**	**A1 (157)**	**A2**	**A3**	**A4 (145)**	**A5**	**A6 (142)**	**A7**	**A8 (17)**	**A9 (17)**	**A10 (158)**	**A11 (159)**	**A14 (156)**	**B**	**P (160)**	**G**
Normal epithelium	N.E.	N.D.	N.D.	expression at very low level	N.D.	N.E.	N.D.	expression at very low level	protein expression verified by IHC and/or WB	expression at very low level	expression at very low level	protein expression verified by IHC and/ or WB	N.D	N.E.	N.D.
Preneoplastic lesions	N.D.	N.D.	N.D.	expression at very low level	N.D.	protein expression verified by IHC and/ or WB	N.D.	protein expression verified by IHC and/or WB	protein expression verified by IHC and/or WB	N.D.	N.D.	N.D.	N.D.	protein expression verified by IHC and/or WB	N.D.
Neoplastic primaries	N.D.	N.D.	N.D.	stagedependent overexpression	N.D.	stage-dependent overexpression	N.D.	overexpression compared to normal tissue	overexpression compared to normal tissue	protein expression verified by IHC and/or WB	N.D.	N.D	N.D.	stagedependent overexpression	N.D.
Metastatic lesions	N.D	N.D.	N.D.	stagedependent overexpression	N.D	stage-dependent overexpression	N.D.	overexpression compared to normal tissue	overexpression compared to normal tissue	overexpression compared to normal tissue (161)	overexpression compared to normal tissue (161)	N.D.	N.D.	stagedependent overexpression	N.D.

Abbreviations: IHC, immunohistochemistry; N.D., no data available; N.E., no expression; WB, Western blotting

#### 3.3.1. S100 Proteins as Biomarkers for Diagnosis and Staging of Colorectal Carcinoma?

CRC development is the result of accumulating genetic and epigenetic alterations that transform normal epithelial cells into invasive adenocarcinomas. This event may either be triggered by exogenous stimuli (eg, high alcohol consumption, smoking and/or high body weight), or chronic intestinal inflammation. Besides these initiators, CRC may also arise in the context of hereditary origins. Despite improved screening and treatment strategies, CRC remains the third leading cause of cancer-related deaths in the world. Therefore, identifying novel biomarkers for the early diagnosis of CRC which will help to improve patients´ outcomes is essential. Examining S100 protein expression has gained a great deal of attention, since these proteins have been implicated in tumor development, metastasis and, in some cases, even chemoresistance. In addition to tests based on analyzing blood or serum samples, S100 proteins may potentially be used as additional markers for tagging patients bearing pre-malignant adenomas of the colorectum. Analysis may ideally be performed on biopsies obtained following a colonoscopy. This may also be conducive in the development of an improved screening protocol. S100A4 (metastasin) is a target gene of the Wnt/ß-catenin pathway, which is constitutively active in most CRC cases. Overexpression was shown to be associated with lymph node metastasis, advanced TNM stage, and poor outcomes (ie, increased recurrence and decreased overall survival), but not Duke subtypes of CRC (99-101). Thus, it was suggested that S100A4 may be necessary for dissemination to distant metastatic sites rather than for local tumor invasion, and it could possibly be a good biomarker for metastasis and prognosis (102). Boye et al. have identified differences in CRC prognosis in terms of cellular localization. Nuclear S100A4 expression was a negative predictor of metastasis- free and overall survival, whereas cytoplasmic S100A4 was not associated with patient outcomes (103). On the contrary, circulating S100A4 is not suitable as an ideal tumor marker, since red blood cells and mononuclear cells also contain S100A4. This can lead to false positive plasma levels in hemolytic samples (104). Whether S100A4 is a useful biomarker or not for CRC has to be carefully re-evaluated. More consistency exists for S100A14, whose expression is inversely correlated with S100A4. Epithelial S100A14 expression was found to be associated with lower invasiveness, metastatic potential and better differentiation, compared to S100A4+ cases (105). Interestingly, reduced expression of S100A14 tends to co-occur with increased S100A4 expression in CRC cell lines and tissue specimens. Therefore, S100A14 is a useful prognostic marker for CRC patients (105). Likewise, S100A6 (calcyclin) might function as a valuable biomarker for the early diagnosing of colonic transformation. It was the first S100 protein to be specifically related to the state of cellular proliferation. S100A6 has two EF-hand motifs. Although S100A6 protein levels can be found in normal colonic mucosa, their expression is dramatically increased during the course of malignant transformation (106). S100A6 is preferentially expressed in the G1 phase of the cell cycle. It has been implicated in regulating cell growth and proliferation via interaction with various ligands, eg, Ca2+/phospholipid-binding proteins of the annexin family and glyceraldehyde-3-phosphate dehydrogenase (107, 108). Besides these quantitative changes, Stulik et al. have also identified differential isoforms in normal and malignant colonic mucosa (107). Clinically, S100A6 expression is significantly correlated with two parameters; lymphatic permeation and Duke’s tumor status (107). S100A8 and S100A9 proteins, usually occurring as the heterodimeric complex, calprotectin, are changed significantly at the early stages of cancer. Plasma levels of S100A8 and S100A9 increase appreciably in patients with colorectal adenomas and established CRCs (109). Therefore, these proteins proteins might, in combination with other serum markers, serve as candidates for serological biomarkers for a CRC diagnosis (110). However, they have not proved effective as a candidate stool marker for detecting CRC. In a clinical study of 77 CRC patients, levels of fecal S100A9 were significantly higher than in the controls, but S100A8 did not show any difference. With regard to sensitivity and specificity, there was no benefit in analyzing S100A9, compared to the conventional fecal occult blood test (111).

Serum S100B protein concentration in stage II-IV melanoma is a reliable prognostic marker. However, no data exists for circulating S100B levels in CRC patients. A recent study found that overexpression within tumors is a more important predictor than merely a conventional pathological variable (112). As determined by immunohistochemistry, S100B overexpression, together with the presence of vascular and perineural invasion, as well as a high postoperative CEA level, was found to be an independent predictor of postoperative early relapse (112). On the contrary, positive S100B protein expression was only found in 6% of non-early relapsed CRC patients. Thus, determining the S100B expression status would probably help to identify patients with a high-risk for early relapse. These patients could then be monitored more intensively and treated accordingly. S100P expression is not restricted to neoplastic cells, but it is also detectable in various normal cell types (eg, enterocytes and epithelial cells). It is therefore not expected to be useful for diagnostic and therapeutic applications (113). Nevertheless, a recent study proposed the likelihood of predicting CRCassociated hepatic metastasis by examining S100P-expression (114). This conclusion follows the observation of increased expression levels during disease progression. Further studies are needed to show if S100P determination will be clinically useful.

#### 3.3.2. S100 Expression and Chronic Inflammation – a Direct Link Towards Tumor Progression?

Several S100 protein family members are differentially expressed in inflammatory bowel diseases, ie, ulcerative colitis and Crohn’s disease, and these have been implicated in inflammation-induced colorectal carcinogenesis (115). Brentnall et al. detected increased expression profiles of S100P, S100A6, S100A11, and S100H during the course of malignant transformation (116). On the other hand, S100A8, S100A9, S100A10, and S100A4 displayed heterogeneous expression patterns in ulcerative colitis patients showing neoplasia (116). Another study proposed that S100A8/A9 expression is an early step in neoplastic transformation during inflammation-associated carcinogenesis. Accordingly, immunohistochemical studies identified S100A8 and A9 expressing macrophages and polymorphonuclear leukocytes along the invasive margin of CRC specimens (109). In normal and chronically inflamed colon mucosa, diffuse staining is usually ob served. Whether this can be seen as an immunological defense mechanism, helping local tumor growth control by chemotactically attracting immune cells to the local microenvironment, or rather forces tumor invasion and metastasis by keeping activated immune cells away from the central tumor burden, is largely unknown. However, the fact that (cytotoxic) T cells can usually be largely found at the invasive margins of CRCs, but only rarely within tumors, hints towards the latter hypothesis. As stated earlier, S100 proteins interact functionally with RAGE. The S100–RAGE signaling pathway plays an important role in linking inflammation and cancer, as well as in tumor cell survival and malignant progression (117). RAGE-deficient tumors are characterized by accelerated apoptosis, reduced NF-κB activation and significantly impaired proliferation (118). Further studies are needed to understand the significance of S100–RAGE interaction in inflammation-induced carcinogenesis in order to assess its appropriateness as a potential target for therapeutic strategies.

#### 3.3.3. S100 Protein Expression in Primary CRCs, Local and Distant Metastases as Well as Possible Implications for Therapeutic Approaches

Local and in particular, distant metastases, are the principal causes of tumor-related complications and death. A common site of metastases derived from the colon following its spread into regional lymph nodes, is the liver. In addition, the presence or absence of lymph node metastasis determines the particular treatment regimen. Metastases are involved in tumor initiation and promotion, uncontrolled proliferation, angiogenesis, invasion, intra- and extravasation, as well as colony formation at the liver site (119). In recent years, several S100 proteins have gained interest because of their involvement in CRC-associated liver metastasis. Despite the well known contribution of S100A4 in mediating CRC progression and driving metastasis, the exact mechanism of how it interacts with other intracellular proteins and exerts pro-metastastic effects remains elusive. Two other S100 family members, namely S100A2 and B were shown to interact with the tumor-suppressor p53 at the transcriptional level and down-regulate wildtype p53 (50). This may drive tumorigenesis. Thus, S100A4 was thought to interact physically and in a regulatory manner with p53, too. However, recent in vitro data indicate that although S100A4 and p53 are present in the same cells, no reciprocal regulatory mechanisms exist (55, 101). Irrespective of the intracellular p53 status, S100A4 levels increase parallel to the number of epithelial cells in the S phase (107). In this regard, calcimycin, a calcium ionophore and transcriptional inhibitor of S100A4, restricts cell motility in CRC cells and inhibits metastasis formation in the intrasplenic HCT116 xenograft mouse model (120). The very same research group also identified that niclosamide, which is an established antihelminthic drug, may act as a potential therapy against S100A4-mediated metastatic colon tumors (121). In addition, a very recent study provided evidence for the suppression of cell growth, migration and invasiveness by specifically silencing S100A4. Metastasis- related genes MMP9, MMP10 and CDH11 were found to be down-regulated after S100A4 suppression, thus indicating a specific association of S100A4 with multiple molecules (122). Likewise, chemosensitization towards methotrexate was achieved by silencing S100A4 in HT-29 CRC cells, which normally do not respond to this cytostatic drug (123). Comparable results were obtained for other chemotherapeutics, suggesting that S100A4 expression can be considered as a biomarker of resistance (124). By 1998, Nakamura and Takenaga had already described the functional mechanisms underlying S100A4 expression in CRCs. They found that hypomethylation of the second intron triggers its expression (125). This supports the emerging concept that hypomethylation may play a role in the up-regulation of genes during later stages of tumorigenesis. Co-expression of S100A6 and S100A11, allows for the discrimination between; primary CRC, liver metastasis, and primary HCC, as well as between CRC and HCC (119). S100A6 and A11 regulate intracellular activities such as; cell growth and motility, cell-cycle progression, transcription, and cell differentiation. Both have been found in epithelial tumors and are linked to metastasis (106). Of particular interest is the finding that S100A6, but not S100A11, is a potential candidate to distinguish between CRC-derived liver metastasis and primary HCC, which is currently a complicated procedure (119). However, S100A11 has just been identified as a valuable marker for regional lymph node metastasis. Immunohistochemical S100A11 expression is significantly correlated with the nodal status, but not tumor depth or grading (126). No studies have so far analyzed the biological functions of S100A6 and/or A11 on chemoresponsiveness.

S100A10 is a plasminogen receptor and this has been suggested as being a promoter of angiogenesis and tumor metastasis. S100 deficiency is associated with early cessation of tumor growth in vivo and this is thought to be due to reduced recruitment of tumor-promoting macrophages to the tumor site (127). Despite lacking firm clinical data, it has been shown that S100A10 silencing decreases plasminogen-dependent invasiveness in a CRC cell line (128). Of particular interest from a therapeutic point of view, is the finding that intracellular S100A10 protein expression levels correlate with sensitivity of CRC cells to oxaliplatin, but not 5-FU or irinotecan (129, 130). Oxaliplatin, a third-generation platinum compound, is commonly used as a combination therapy (=FOLFOX) for CRC patients. However, approximately half of these patients will be unlikely to benefit from this treatment regimen due to chemoresistance (129). Assessing the S100A10 status could thus facilitate prediction of individual responsiveness towards platinum-based chemotherapies. Comparably little is known about the functional contribution of S100B to CRC progression and metastasis. S100B binds to different oligomeric forms of p53 and regulates its activity. S100B binding to p53 disables its biological function as a tumor suppressor and probably drives carcinogenesis. These activities are mediated via interactions with target proteins such as; annexins, cytosolic phospholipase A2, the Ca2+ release channel of the sarcoplasmic reticulum, and myosin (131). In Taiwanese CRC patients, liver metastasis is correlated with S100B overexpression (132).

Besides pancreatic and breast cancer, S100P, originally isolated from a placenta, is overexpressed in tumors of the colorectum. Here, elevated S100P levels have been found in epithelial cells (113, 133). S100P is linked to immortalization, malignancy, hormone-independency, chemoresistance and poor clinical outcomes (134). S100P expression, among other genes, seems to be essential for CRC liver metastasis (114). In a similar vein, S100P expression correlates with two other metastasis-inducing proteins, S100A4 and osteopontin. Recently, Chandramouli et al. shed light on the regulatory mechanisms of this pro-tumorigenic molecule. They showed that S100P is significantly up-regulated by prostaglandin E2 (PGE2) via the EP4 receptor, which is overexpressed in epithelial CRC cells. Hence, pro-tumorigenic PGE2 stimulation activates downstream signals (ie, ERK phosphorylation and NF-κB activation) and this influences morphology, colony growth, tumor cell motility, and chemoresponsiveness (135).

Elevated NF-κB activity is linked to increased chemoresistance (136, 137). Bertram et al. observed doxorubicin resistance in S100P-expressing CRC cells (138), while S100P absence or knockdown inhibits CRC cell growth, migration and invasion in vitro, as well as tumor growth and liver metastasis in a xenograft nude mouse model in vivo (139, 140). S100P regulation by the PGE2/EP4 receptor signaling pathway may therefore constitute a feedback regulatory mechanism by which tumor cells perpetuate growth and migration during carcinogenesis.

### 3.4. Hepatocellular Carcinoma

Tumors in the liver arise due to; chronic liver injury, inflammation, and hepatocyte proliferation, and this can be provoked by several causes such as; chronic hepatitis B and C viral infections, chronic alcohol consumption, and aflatoxin B1–contaminated food (141). Screening for HCC has so far not improved the prognosis of this condition. Hence, novel markers for early disease detection and therapy decision making are warranted. Here, we discuss the current value on S100 protein detection within HCC.

#### 3.4.1. S100 Proteins as Biomarkers for Hepatocellular Carcinoma Diagnosis and Staging?

S100A6 is very specific marker for malignancy, since expression is absent in normal, non-cancerous liver tissue. This signal transduction molecule forces cell proliferation, differentiation, migration, and cytoskeletal dynamics. S100A6 expression, particularly when associated with osteopontin, another pro-metastatic protein and high a-fetoprotein levels, are associated with poor tumor differentiation. It may therefore be a promising diagnostic marker and therapeutic target for HCC (142). Comparing S100A9 expression in 70 HCC with non-carcinomatous hepatocytes and bile duct epithelia revealed exclusive immunoreactivity in tumor cells. These findings hint towards a neo-expression in differentiated malignant hepatocytes, which is related to deprived tumor differentiation. However, no correlation exists with regard to vascular invasion (143). Therefore, S100A9 may be involved in initial tumor formation rather than in metastasis.

#### 3.4.2. Detecting S100 Proteins in HCC Cell Lines and Primary as Well as Metastatic Lesions With Possible Implications for Therapeutic Approaches

Very few studies have so far analyzed S100P expression in HCC, despite its well-known association with a variety of neoplastic diseases. Liver cancer cell lines express higher S100P levels than normal non-malignant hepatocytes, which frequently lack S100P. In an in vitro study, Kim et al. showed that endogenous S100P silencing decreases HCC cell growth. The anti-mitogenic effect could be partially attributed to cell cycle deregulation and apoptosis induction. Nevertheless, as opposed to CRCs, silencing of endogenous S100P by siRNA did not alter phosphorylation or protein expression levels of the S100-downstream molecules ERK and NF-κB (110). The functional role of S100P on liver carcinogenesis, therefore, still remains elusive. In situ, S100P expression gradually increases from dysplasia, precancerous lesions, to HCC. Hence, S100P, which is known to act as an aggressiveness factor in HCC cells, may be conducive for HCC formation or progression. S100A6 contributes to cell proliferation and invasion in different ways. These include activin A inhibition and transcription factor 3 activation. This can affect MMP functions (eg, MMP-2) and cytoskeletal reorganization in conjunction with cathepsin D (142). Besides, cellular S100A6 expression is inducible by NF-κB (p65) following TNF-α stimulation (18).

S100A4 expression is associated with epithelial mesenchymal transition (EMT), a tumor-cell invasion process that results in the loss of epithelial characteristics and the acquisition of mesenchymal features. In HCC, S100A4 expression, together with other EMT-related proteins, is synonymous with metastasis as well as decreased diseasefree and overall survival (144). Hence, S100A4-expressing tumor cells have a much stronger metastatic ability than their S100A4-negative counterparts (145). In cholangiocarcinoma cell lines, S100A4-silencing leads to reduced motility, invasiveness, and MMP-9 secretion in vitro. Up to now, S100A4 function has remained largely unknown, as it is still uncertain if S100A4 is just a biomarker of cellular aggressiveness or represents a functional target for therapeutic interventions. S100A8 and S100A9 are NF-κB target genes in HCC cells during inflammation-associated liver carcinogenesis. While both proteins are seldom found in normal liver tissues, their expression increases with malignant transformation and facilitates disease progression. The underlying molecular mechanism has been described as due to activation of ROS-dependent signaling pathways and protection from TNF-a induced cell death (17). Possible consequences for the clinics are still pending.

S100A11 has an EF-hand domain and mediates growth inhibition in normal fibroblasts through p21/Waf1 activation. In an experimental study, comparable effects were described for human HCC cells, in which proliferation was blocked following TGF-ß treatment (66). TGF-ß is an important physiological inducer of apoptosis in HCC cells that exerts its various effects via two transmembrane serine/threonine kinases. This may hint towards another role of S100A11, not only its function as a pro-tumorigenic molecule, but also as a growth inhibitor. These findings fit well with the ambiguous and varying role S100A11 plays between different tumor entities.

#### 3.4.3. Latent Chronic Liver Inflammation and the Relevance of S100 Proteins?

Chronic inflammation represents a high risk factor for the development of various cancers, and S100 proteins have been implicated in many aspects of the interaction between tumor, stromal, and immune cells contributing to the formation of an inflammatory tumor microenvironment (146). Apparently, liver cancer is not related to chronic inflammation; however, persistent viral or bacterial inflammation has been demonstrated to initiate or trigger HCC. The prevalence of chronic hepatitis B (circular DNA virus) and C (positive-strand RNA virus) infection is directly linked to HCC. While HBV integrates into the host genome and has reverse transcriptase activity, HCV does not. Common to both viruses is an immune-mediated inflammatory response that either clears the infection or slowly destroys the liver. Viruses are recognized by the hosts’ immune system as pathogen-associated molecular patterns via TLRs (e.g. TLR 3 and 7). This activates a signal cascade, leading to NF-κB activation, which finally links inflammation and immunity to cancer development and progression. Despite the relevance of S100 proteins in inflammation and cancer, these proteins have rarely been studied in virus-driven HCC. HCV-associated carcinogenesis arises as a consequence of complicating cirrhosis. The process of liver fibrosis following a HCV infection may be accompained by EMT of the bile duct epithelial cells. In situ as well as experimentally, EMT is induced by TGF-ß1, transforming normal HCV infected cells into invasive cells. Moreover, TGF-ß1 induces S100A4 expression upon LPS stimulation of human intrahepatic biliary epithelial cells (147) and elevated levels of S100A4-positive cells are found in all forms of chronic liver disease, including chronic HCV infection, alcoholic liver disease, non-fatty liver disease, hereditary hemochromatosis, and cryptogenic cirrhosis (148). Hence, S100A4 expression is directly linked to inflammation-induced carcinogenesis.

In the case of HBV, HCC is habitually found in non-cirrhotic livers. S100A10 inhibits the HBV Pols’ DNA polymerase activity (149). Besides this, serum S100B seems to be a sensitive marker of endothelial and tissue damage in chronic HCV hepatitis (150). Interestingly, while S100A8/A9 is actively involved in chronic bowel inflammation, this complex is supposed to exert the opposite function in the liver by indirectly suppressing nitrogen oxide overproduction from activated neutrophils and/or macrophages. In a rat model of acute LPS-induced inflammation, the activity of proinflammatory cytokines could be neutralized by intraperitoneal injections (151). Hence, S100A8/A9 is rather more involved in liver regeneration than in promoting inflammation. Further indirect evidence is given by the observation that S100A8/A9 gene expression is induced in virus-infected keratinocytes via TLR3 ligation (152).

## 4. Conclusions and Future Perspectives

The family of S100 Ca2+-binding proteins is closely related to several tumors including those of the colon and liver. In general, S100 overexpression is coupled with poor tumor differentiation, aggressiveness, advanced stage, and metastatic growth. Therefore, S100 expression is considered to be a negative prognosticator for patient’s survival. However, recent data has suggested that this conclusion is too short-sighted and S100 proteins may therefore be regarded as playing both positive and negative roles. Further research is therefore required in order to discriminate the complex expression patterns of S100 proteins in tumor and stromal cells, as well as in their pro-tumorigenic and anti-tumorigenic actions. Answers to these issues are needed, before we can more fully understand the clinical relevance of S100 protein expression within epithelial tumors with regard to their potential applicability as biomarkers, for diagnosis and therapy decisions.
